# Novel Atomic Absorption Spectrometric and Rapid Spectrophotometric Methods for the Quantitation of Paracetamol in Saliva: Application to Pharmacokinetic Studies

**DOI:** 10.4103/0250-474X.42999

**Published:** 2008

**Authors:** M. M. Issa, R. M. Nejem, N. S. El-Abadla, M. Al-Kholy, Akila. A. Saleh

**Affiliations:** Department of Analytical Chemistry, Alaqsa University, P.O.Box 4051, Gaza-76888, Palestine; 1 Department of Organic Chemistry, Alaqsa University, Gaza, Palestine; 2 Department of Analytical Chemistry, Ain Shams University, Cairo, Egypt

**Keywords:** Spectrophotometry, atomic absorption spectrometry, paracetamol, pharmacokinetic, within-day variability, between-day variability

## Abstract

A novel atomic absorption spectrometric method and two highly sensitive spectrophotometric methods were developed for the determination of paracetamol. These techniques based on the oxidation of paracetamol by iron (III) (method I); oxidation of p-aminophenol after the hydrolysis of paracetamol (method II). Iron (II) then reacts with potassium ferricyanide to form Prussian blue color with a maximum absorbance at 700 nm. The atomic absorption method was accomplished by extracting the excess iron (III) in method II and aspirates the aqueous layer into air-acetylene flame to measure the absorbance of iron (II) at 302.1 nm. The reactions have been spectrometrically evaluated to attain optimum experimental conditions. Linear responses were exhibited over the ranges 1.0-10, 0.2-2.0 and 0.1-1.0 μg/ml for method I, method II and atomic absorption spectrometric method, respectively. A high sensitivity is recorded for the proposed methods I and II and atomic absorption spectrometric method value indicate: 0.05, 0.022 and 0.012 μg/ml, respectively. The limit of quantitation of paracetamol by method II and atomic absorption spectrometric method were 0.20 and 0.10 μg/ml. Method II and the atomic absorption spectrometric method were applied to demonstrate a pharmacokinetic study by means of salivary samples in normal volunteers who received 1.0 g paracetamol. Intra and inter-day precision did not exceed 6.9%.

Paracetamol (N-acetyl-p-aminophenol) has been in use as analgesic and antipyretic drug for over 50 years. It has been accepted as a very effective medication for the relief of pain and fever in adults and children^1^. A review of broad scope analytical interest such as chemical, physical and biopharmaceutical properties on paracetamol has been published^2^,^3^. Several spectrophotometric methods have been reported for the determination of paracetamol based on nitration^4^,^5^, oxidation^6^–^8^ and hydrolysis to p-aminophenol followed by diazotization and phenolic coupling 9–13.

The reduction process of iron (III) by diclofenac sodium^14^, salbutamol sulfate^15^, captopril^16^, amoxycillin^17^ and ciprofloxacin^17^ to iron (II) were used to quantify these drugs colorimetrically. The reversible redox system iron (III)/iron (II) was used as indicating aspect for the indirect flow-injection biamperometric determination of paracetamol^18^ and in simultaneous spectrophotometric determination of paracetamol by a differential kinetic method^19^.

Liu and Oka described a spectrophotometric technique for the detection of paracetamol in serum and plasma based on the reduction of ferric 2,4,6-tris(2-pyridyl)-5-triazine, to ferrous 2,4,6-tris(2-pyridyl)-5-triazine complex^20^. The method was specific to compounds that contain a phenolic hydroxyl group located in either Meta or para position on the benzene ring over a linear range from 25 to 400 mg/l^20^. The sensitivity of their method was not adequate to perform a pharmacokinetic study. Also some other drugs which have no phenolic hydroxyl group can reduce iron (III), like diclofenac^14^, captopril^16^ and ciprofloxacin^17^. Diclofenac and captopril are reducing agents due to the presence of aromatic amine (-NH) and thiol (-SH) groups in their structure.

In the present study, three methods were proposed for the determination of paracetamol based on spectrophotometric and atomic absorption spectrometry (AAS) techniques. The spectrophotometric methods (I and II) were based on the direct reduction of iron (III) with paracetamol and the hydrolysis of paracetamol to p-aminophenol, respectively. A Prussian blue color was formed due to the reaction of iron (II) with potassium ferricyanide, whose intensity is proportional to the concentration of paracetamol. P-aminophenol is a reducing agent due to the presence of phenolic hydroxyl and aromatic amine groups. The present study also aimed to develop a novel AAS method for the quantitation paracetamol in salivary samples where the literature reveals no researches cover the determination of paracetamol based on AAS. The AAS method based on the extraction of iron (III) from 6.0 M hydrochloride acid with diethyl ether (probably as the solvated complex [H_3_O(R_2_O)_2_^+^,FeCl_4_^-^]^21^ and the aqueous layer was used for atomic absorption measurement of iron (II).

The spectrophotometric method II and AAS techniques were applied for the determination and assessment of the bioavailability of paracetamol using saliva samples. The pharmacokinetic study of paracetamol by means of saliva was considered feasible, since the ease of samples collection and analysis besides the good correlation between saliva and plasma levels of paracetamol^22^.

## MATERIALS AND METHODS

Paracetamol was supplied by the Middle East pharmaceuticals and Cosmetics Laboratories, Palestine (Kempex BV Holland). Paracetamol tablets, 500 mg, were purchased from the pharmacy, Decamol (Megapharm) and Dexamol (Dexxon). Standard solutions of paracetamol (10 μg/ml) were prepared in deionized water and diluted as required. Potassium ferricyanide solution (1.0 mM) and ferric sulfate solution (1.0 mM) was also prepared in deionized water. All chemicals and reagents used were of analytical grade.

A spectro 20 D plus, Labomed Inc. USA spectrophotometer with 1.0 cm rectangular glass cell was used for all spectrophotometric measurements. Atomic absorption measurements were performed using Perkin-Elmer AAS, model A Analyst 100 spectrophotometer equipped with an iron hollow-cathode lamp, under the following conditions: wave length, 302.1 nm, slit-width 0.2 nm, lamp current 30.0 mA, air flow rate and acetylene flow rate are adjusted at the standard conditions.

### Spectrophotometric method I:

Six different portion of paracetamol standard concentrations (0, 1.0, 2.0, 4.0, 6.0, 8.0 and 10.0 μg/ml), 2.5 ml each, were pipetted into a series of 5 ml measuring flasks. To each flask, 1 ml of ferric sulfate solution (1.0 mM) and 1.0 ml of potassium ferricyanide solution (1.0 mM) were added and completed with deionized water. Absorbances were recorded at 700 nm after 24 min^16^.

### Spectrophotometric method II:

Six different portion of paracetamol standard concentrations (0, 0.2, 0.4, 0.8, 1.2, 1.6 and 2.0 μg/ml), 2.5 ml each portion, were pipetted into a series of 5.0 ml measuring flasks. To each flask, 0.5 ml of 1.0 M HCl solution was added, followed by 1.0 ml of ferric sulfate. The mixtures were heated using a boiling water bath at 100° for 10 min. After cooling, 1.0 ml of potassium ferricyanide was added and diluted with water. Absorbances were recorded at 700 nm after 24 min^16^.

### AAS method:

Six different portion of paracetamol standard concentrations (0, 0.1, 0.2, 0.4, 0.6, 0.8, and 1.0 μg/ml), 2.5 ml each, were pipetted into a series of 10 ml measuring flasks. To each flask, 0.5 ml of 1.0 M HCl solution was added, followed by 1.0 ml of ferric sulfate. The mixtures were heated using a boiling water bath at 100° for 10 min and after cooling 4.0 ml of 12.0 M HCl solution was added. Iron (III) was extracted with three 25.0 ml portions of diethyl ether. The aqueous layer was aspirated into the air-acetylene flame, and the absorbance of iron (II) was measured at 302.1 nm.

Saliva standard solutions for calibration curve were prepared by adding (0, 0.1, 0.2, 0.4, 0.6, 0.8, 1.0, 1.2, 1.6 and 2.0 ml) of 2.5 μg/ml of paracetamol to 0.25 ml drug free saliva using micropipette and complete the volume to 2.5 ml. The final concentrations of paracetamol each saliva sample were 0, 0.1, 0.2, 0.4, 0.6, 0.8, 1.0, 1.2, 1.6 and 2.0 μg/ml.

### Extraction of saliva samples:

The extraction is performed based on the procedure described by Dordoni *et al*.^23^ and Spooner *et al*.^24^. An amount of 1.0 g Na_2_SO_4_ and 10 ml of diethyl ether were added to 0.25 ml saliva aliquot or standard and mixed thoroughly. The diethyl ether layer was separated and evaporated. The residue was dissolved in 2.5 ml water and determined by spectrophotometric method II or AAS method.

### Analysis of paracetamol from tablet dosage form in pharmaceutical preparations:

Twenty tablets of paracetamol (500 mg paracetamol active ingredient) were accurately weighed and powdered, Decamol (Megapharm, Palestine) and Dexamol (Dexxon, Israel). A portion equivalent to 50 mg was dissolved in distilled water and analyzed by the recommended procedures.

### Pharmacokinetic application:

The pharmacokinetic study has been approved by the Ministry of Health, PNA. Five healthy female volunteers, with ages ranging from 20-24 years and weights 55-75 kg, were enrolled in the pharmacokinetic study. In a randomized two-way crossover design, each subject received 1.0 g dose of paracetamol (2 tablets) on an empty stomach and overnight fast. Tablets were administered with 200 ml of water. No food was allowed for 4 h after which a light standard lunch was served. The volunteers were instructed to drink water regularly during the study to keep saliva flow. Three ml saliva samples were collected before drug administration as Blank and at 0.25, 0.5, 0.75, 1, 1.25, 1.5, 1.75, 2, 4, 6 and 8 h, a wash period of 7 days separated each two consecutive phases of the crossover. Saliva samples were centrifuged for 10 minutes and 0.25 ml of the supernatant was taken and analyzed using spectrophotometric method II or AAS method.

## RESULTS AND DISCUSSION

**Fig. 1:**
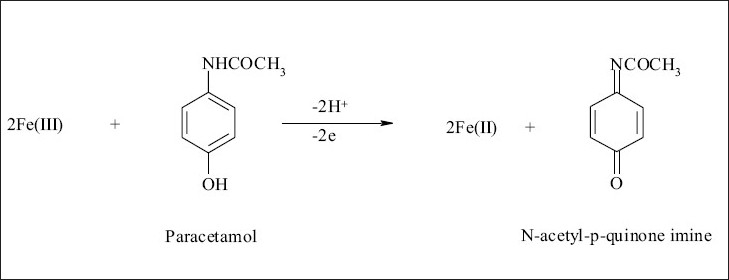
Reaction of paracetamol with iron (III) in spectrophotometric method I.

Oxidation of paracetamol with iron (III) followed by potassium ferricyanide to form a Prussian blue color whose intensity is proportion to the concentration of paracetamol was performed. Paracetamol was quantitatively oxidized at room temperature (25°) without any significant effect of pH. It was found that keeping paracetamol concentration constant and altering the concentration of iron (III), caused an increase in the absorbance up to mole ratio of 2:1 iron (III) and paracetamol, respectively. The probable reaction as shown in fig. 1, N-acetyl-p-quinone imine was only produced by the oxidation of paracetamol, 2,2-dihydroxy-5,5’-diacetylaminobiphenyl is formed only in alkaline medium^25^,^26^.

**Fig. 2:**
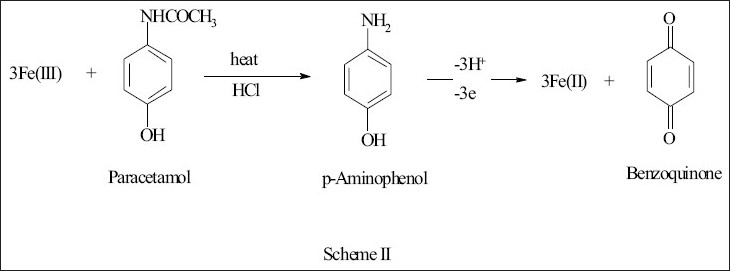
Oxidation and hydrolysis of paracetamol in spectrophotometric method II and AAS.

Paracetamol was hydrolyzed in acidic medium using boiling water bath to give p-aminophenol which reduced iron (III). Heating intervals and pH were optimized to ensure complete hydrolysis and oxidation. Different heating intervals were investigated where 10 min were found to be enough for the complete reaction. It was also observed that the absorbance remained constant in the pH range 1.0-3.0. Therefore, all the studies were performed at pH 1.0. The stoichiometry of the reaction was established as in method I and the iron (III) to paracetamol mole ratio was found 3:1 (fig. 2).

Different aliquots of the standard iron (III) were dissolved in 6.0 M HCl solutions and each sample was extracted with three 25 ml portions of diethyl ether. Iron (III) in the aqueous layer was determined colorimetrically using KSCN at 480 nm or by AAS method at 302.1 nm. The overall extraction efficiency was approximately 100% and 99.99% using colorimetric and AAS method, respectively.

Detection limit (LOD) of the proposed methods was determined by analysis of the peak baseline noise in five blank samples, which was considered as three times the variation in measured response. The LOD were 0.12, 0.05 and 0.026 μg/ml for spectrophotometric method I and II and AAS method, respectively. The estimated limit of quantification (LOQ) was calculated as ten times the variation in measured response. The LOQ were 0.40, 0.20 and 0.10 μg/ml for spectrophotometric method I and II and AAS method, respectively. The LOQ were confirmed for saliva using calibrators with nominal concentration of 0.20 and 0.10 μg/ml. The sensitivity was determined as the concentrations that gave an absorbance reading of 0.0044. The sensitivity of the proposed methods were 0.05, 0.022 and 0.012 μg/ml methods I, and II and AAS, respectively.

Typical calibration curves were constructed for the three proposed methods based on linear regression analysis of absorbance versus concentration. The slope, intercept, sensitivity and correlation coefficient were summarized (Table 1). Linear responses were displayed in the range 1.0-10, 0.2-2.0 and 0.1-1.0 μg/ml for paracetamol using spectrophotometric method I, and II and AAS method, respectively. The molar absorptivities were 1.35×10^4^ and 2.72×10^4^ l/mol.cm for paracetamol using spectrophotometric method I and II, respectively.

**TABLE 1:** RESPONSE CHARACTERISTICS OF THE PROPOSED METHODS

Parameters	Spectrophotometric method		AAS
		
	I	II	
λ (nm)	700	700	302.1
pH	-	1	6
Molar absorptivity (L/mol.cm)	1.35×10^4^	2.72×10^4^	-
LOD (μg/ml)	0.12	0.05	0.026
LOQ (μg/ml)	0.40	0.20	0.10
Sensitivity (μg/mL)	0.05	0.022	0.012
Linear response (μg/ml)	1.10	0.2-2	0.1-1
RSD (n=5)	3.86	5.0	2.55
Regression equation			
Slope	0.089±0.007	0.155±0.012	0.40±0.033
Intercept	0.011±0.001	0.049±0.003	0.025±0.002
Correlation coefficient (R)	1	0.9920	0.9982

Five replicate measurements were performed using the three proposed methods. The relative standard deviations (RSD) were 3.86, 5.0 and 2.55 for spectrophotometric method I, and II and AAS method, respectively. The proposed methods were applied for the recovery of paracetamol in its dosage forms. The obtained results using the proposed methods are in good concordance with the standard method of British pharmacopoeia^27^. The comparison through using t- and f- statistical tests confirm the high accuracy and precision of the proposed methods (Table 2).

**TABLE 2:** ASSAY OF PARACETAMOL IN BULK AND DOSAGE FORMS BY THE PROPOSED METHODS AND OFFICIAL METHOD^27^

Sample	Recovery % ± SD based on different methods^a^			
	
	Spectrophotometric method		AAS	Standard^27^ method
			
	I	II		
Paracetamol bulk	100.2±0.31	98.5±0.22	99.6±0.44	99.7±0.47
Decamol (500 mg/tablet)	98.8±0.52	100.8±0.71	98.9±0.51	99.9±0.74
Dexamol (500 mg/tablet)	99.2±0.47	99.6±0.18	99.4±0.69	100.1±0.55
t-test / f-test				
Paracetamol bulk	0.29 / 2.3	1.53 / 1.7	1.12 / 2.6	
Decamol (500 mg/tablet)	1.33 / 2.9	1.24 / 2.8	1.34 / 3.8	
Dexamol (500 mg/tablet)	1.85 / 1.3	1.87 / 3.2	1.67 / 1.6	

a Average of five determination, t (n=5) = 2.776, f (5,5) = 6.39.

Within-day variability of the proposed methods for the analysis of paracetamol were determined by repeating the analysis of three quality control samples at low, medium and high concentrations on the same day. The results indicate that these methods are reproducible within the same day (Table 3). Between-day variability of the proposed methods were determined by repeated analysis of three quality control samples at low, medium and high concentration on three different days. The results are shown in Table 3. This data proves the reproducibility of the proposed methods within different days.

**TABLE 3:** WITHIN- AND BETWEEN-DAY VARIABILITY OF PROPOSED METHODS FOR PARACETAMOL DETERMINATION

Paracetamol (μg/ml)	Within-day variability (±SD^a^)			RSD	SAE^b^	CL^c^
				
	Spectrophotometric					
				
	I	II	AAS			
1	1.08±0.088	-	-	8.15	0.039	1.08±0.109
6	5.9±0.324	-	-	5.49	0.145	5.9±0.402
10	10.18±0.50	-	-	4.91	0.224	10.18±0.621
0.2	-	0.2±0.012	-	6.00	0.005	0.2±0.015
0.8	-	0.84±0.062	-	7.38	0.028	0.84±0.077
2.0	-	1.95±0.056	-	2.86	0.250	1.95±0.700
0.1	-	-	0.1±0.007	7.00	0.003	0.1±0.009
0.6	-	-	0.59±0.026	4.40	0.120	0.59±0.032
1.0	-	-	1.01±0.020	1.98	0.009	1.01±0.025
Between-day variability (±SD^a^)					
1	1.1±0.130	-	-	11.82	0.058	1.1±0.161
6	6.2±0.267	-	-	4.30	0.119	6.2±0.330
10	10.6±0.496	-	-	4.68	0.222	10.6±0.616
0.2	-	0.22±0.012	-	5.45	0.005	0.22±0.139
0.8	-	0.85±0.052	-	6.12	0.023	0.85±0.640
2.0	-	1.96±0.053	-	2.70	0.023	1.96±0.640
0.1	-	-	0.11±0.007	6.38	0.003	0.11±0.008
0.6	-	-	0.61±0.020	3.30	0.009	0.61±0.025
1.0	-	-	1.01±0.026	2.57	0.012	1.01±0.033

a Average of five determinations

b SAE, standard analytical error

c Confidence limits (CL), 95% and 4 degree of freedom

The effect of the presence of common excipients such as dextrose, glucose, saccharine sodium, starch, talc and magnesium stearate were investigated. There were insignificant interferences from these excipients. The most of drugs (ascorbic acid, caffeine, salbutamol, diclofenac sodium, amoxycillin and ciprofloxacin) and metabolites were reduced by iron (III) and paracetamol should therefore be extracted and separated before applying the methods.

**Fig. 3:**
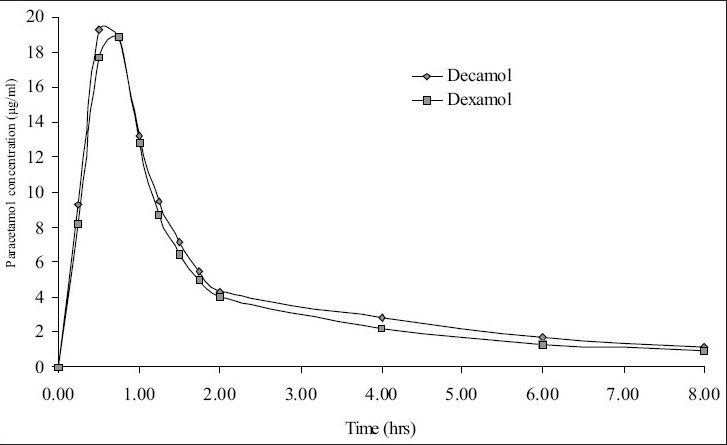
Mean saliva concentration curves using spectrophotometric method II. Decamol (-□-), Dexamol (-◇-).

**Fig. 4:**
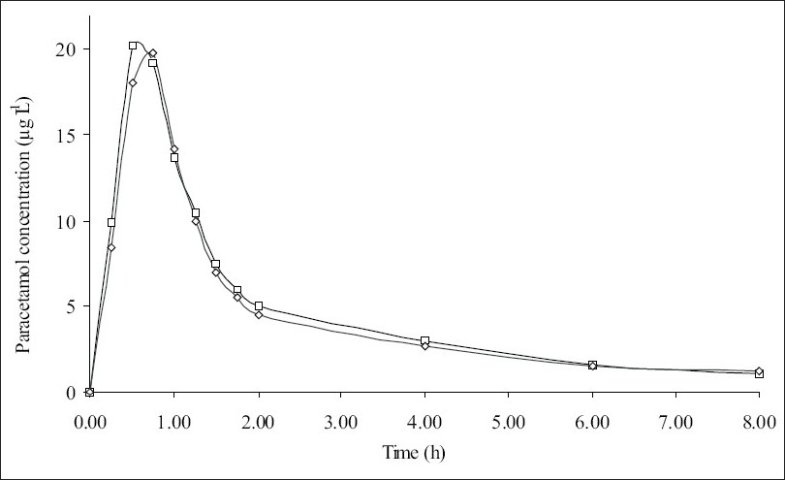
Mean saliva concentration curves using AAS. Decamol (-□-), Dexamol (-◇-).

Five healthy female volunteers were administrated 1 g of Dexamol and Decamol separately. The recovery investigations of standard saliva samples spiked with paracetamol are shown in Table 4. The recovered concentrations of paracetamol using the three proposed methods are highly correlated to the spiked amounts of paracetamol. The mean saliva concentration-time curves based on spectrophotometric method II and AAS are shown in figs. 3 and 4, respectively. Paracetamol saliva concentration did not show any significant difference between the two brands at each sampling.

**TABLE 4:** RECOVERY OF SPIKED PARACETAMOL IN THE CONTROL SALIVA SAMPLES

Saliva (ml)	Paracetamol Spiked (μg/ml)	Recovery based on spectrophotometric Method II^a^		Recovery based on AAS Method^a^	
			
		(μg/ml)	%	(μg/ml)	%
0.25	0.1	-	-	0.105±0.002	105
0.25	0.2	0.21±0.003	105	0.19±0.005	95
0.25	0.4	0.39±0.007	97.5	0.38±0.008	95
0.25	0.6	-	-	0.59±0.012	98
0.25	0.8	0.82±0.015	102	0.820±0.013	103
0.25	1.0	-	-	1.01±0.033	101
0.25	1.2	1.18±0.041	98	-	-
0.25	1.6	1.56±0.053	97.5	-	-
0.25	2.0	1.88±0.067	94	-	-

a Average of five determination

The pharmacokinetic parameters for the two brands, maximum salivary concentration (Cmax, μg/ml), time to maximum saliva concentration (T_max_, h) and area under the saliva concentration-time curve (AUC_0-8_ μg/ml h) are summarized (Table 5). The mean values of AUC_0-8_ oral bioavailability of Decamol tablet was 103% relative to Dexamol tablet (innovator brand) in each described assay. Thus, the bioavailability of the two brands was not significantly different; therefore the two products are considered bioequivalent.

**TABLE 5:** PHARMACOKINETIC PARAMETERS IN HEALTHY FEMALE VOLUNTEERS

Parameters	Spectrophotometric method II		AAS method	
		
	Dexamol tablet	Decamol tablet	Dexamol tablet	Decamol tablet
C_max_ (μg/ml)	18.9	19.3	19.1	20.2
T _max_ (h)	0.6	0.5	0.6	0.5
T_½_ (h)	2.76	2.55	2.82	2.69
AUC_0-8_ (μg/ml.h)	34.7	35.9	35.2	36.3
% AUC	100	103	100	103

Pharmacokinetic parameters after the administration of 1.0 g paracetamol in five healthy female volunteers

All the proposed methods are simple, rapid, accurate, and exhibit higher sensitivity compared to Zarei *et al* and Liu and Oka methods^19^,^20^ and the AAS is a novel method. The extraction of paracetamol from saliva into diethyl ether improves the specificity of the assay. The statistical parameters and the recovery study data clearly indicate the reproducibility and accuracy of the methods. The proposed methods are suitable for the analysis of paracetamol in its pharmaceuticals formulations without any interference from the exipients normally found in commercial preparations. Spectrophotometric method II and AAS method are highly sensitive for the application in the pharmacokinetic studies.
